# ^18^F-FDG PET/CT-based early treatment response evaluation of nanoparticle-assisted photothermal cancer therapy

**DOI:** 10.1371/journal.pone.0177997

**Published:** 2017-05-24

**Authors:** Kamilla Norregaard, Jesper T. Jørgensen, Marina Simón, Fredrik Melander, Lotte K. Kristensen, Pól M. Bendix, Thomas L. Andresen, Lene B. Oddershede, Andreas Kjaer

**Affiliations:** 1 Dept. of Clinical Physiology, Nuclear Medicine & PET and Cluster for Molecular Imaging, Rigshospitalet and University of Copenhagen, Copenhagen, Denmark; 2 Center for Nanomedicine and Theranostics, DTU Nanotech, Technical University of Denmark, Lyngby, Denmark; 3 Niels Bohr Institute, University of Copenhagen, Copenhagen, Denmark; Brandeis University, UNITED STATES

## Abstract

Within the field of nanoparticle-assisted photothermal cancer therapy, focus has mostly been on developing novel heat-generating nanoparticles with the right optical and dimensional properties. Comparison and evaluation of their performance in tumor-bearing animals are commonly assessed by changes in tumor volume; however, this is usually a late-occurring event. This study implements 2-deoxy-2-[F-18]fluoro-D-glucose positron emission tomography imaging to perform early evaluation of the treatment outcome of photothermal therapy. Silica-gold nanoshells (NS) are administered intravenously to nude mice bearing human neuroendocrine tumor xenografts and the tumors are irradiated by a near-infrared laser. The animals are positron emission tomography scanned with 2-deoxy-2-[F-18]fluoro-D-glucose one day before and one day after treatment. Using this setup, a significant decrease in tumor uptake of 2-deoxy-2-[F-18]fluoro-D-glucose is found already one day after therapy in the group receiving NS and laser treatment compared to control animals. At this time point no change in tumor volume can be detected. Moreover, the change in tumor uptake, is used to stratify the animals into responders and non-responders, where the responding group matched improved survival. Overall, these findings support the use of 2-deoxy-2-[F-18]fluoro-D-glucose positron emission tomography imaging for preclinical and clinical evaluation and optimization of photothermal therapy.

## Introduction

Nanoparticle-assisted photothermal therapy is a technique that exploits the strong light-to-heat conversion of plasmonic nanoparticles when irradiated with resonant light[1–3]. The therapy is particularly well-suited for cancer because tumors in general are known to have leaky vasculature that enables nano-sized drugs and particles to passively accumulate in the tumor tissue when injected into the bloodstream[4–6]. The leaky vasculature is known as the enhanced permeability and retention (EPR) effect and is a consequence of the chaotic vascular growth during tumor angiogenesis. The photo-induced heating of the nanoparticles is strictly local and can be triggered by an external light source minimizing adverse effects on surrounding healthy tissue[7]. Furthermore, using near-infrared (NIR) light as nanoparticle-excitation source reduces unspecific tissue heating due to the high tissue transparency in this window[8,9].

Since the first implementation of nanoparticle-assisted photothermal therapy[10], researchers have put much effort into developing novel NIR resonant nanoparticles that provide good heat generation as well as accumulate efficiently in tumors[1,2,11–13]. One of the most promising candidates is silica-gold nanoshells (NS), a class of NIR absorbing nanoparticles that consist of a silica core surrounded by a thin gold shell. It has been shown extensively in literature that NS accumulate passively in tumor tissue, as well as have the ability to generate sufficient heat upon NIR irradiation to inflict severe tumor damage in mice[1,10,14–16]. This combined with the biocompatibility and inertness of gold, has resulted in their inclusion in FDA approved early clinical trials for photothermal treatment of head and neck tumors (NCT00848042)[17], prostate tumors (NCT02680535)[18], as well as primary and metastatic lung tumors (NCT01679470)[19]. Furthermore, the latest clinical safety profiles are promising showing no indication of toxicity[20,21]. In fact, despite the wide range of heat-generating nanoparticles presented in literature, to our knowledge, NS are the only nanoparticle for photothermal therapy that has progressed to clinical trials.

Although the photothermal performance and tumor uptake of the nanoparticles play a huge role in the overall treatment efficiency, and rightfully should receive much attention, little focus has been put on optimizing the treatment protocol for the best therapeutic outcome in a pre-clinical setup[13]. Treatment response evaluation has commonly been based on monitoring morphological tumor changes that is a slow and late-occurring event, or on optical imaging of bioluminescent cancer cells that is clinically irrelevant. Alternatively, medical imaging techniques such as positron emission tomography (PET) and magnetic resonance imaging (MRI) that can reveal functional changes in the tumor shortly after therapy hold great promise, as they improve the ability to predict outcome and modify the forward going therapy at an early stage. Furthermore, these imaging techniques can also be used to visualize and track nanoparticles in real-time, provided they are labeled with radioisotopes or MR contrast agents, that give valuable information about their path and fate after injection[12,22–26].

In relation to photothermal therapy, PET imaging has mostly been used for real-time tracking of radiolabeled nanoparticles but there also exist a few pre-clinical studies where the tracer 2-deoxy-2-[F-18]fluoro-D-glucose (^18^F-FDG) has been applied to quantify treatment effect after photothermal therapy[2,12,27]. ^18^F-FDG is a radioisotope labeled glucose analogue that is taken up by the cell via the same pathway as glucose but becomes trapped intracellularly, as once phosphorylated it cannot be further metabolized. Thus, ^18^F-FDG is a marker for metabolic activity and it has been widely used as a PET tracer in clinic for diagnosing and staging cancer[28]. Recently we showed, using small animal PET imaging, that a reduced uptake of ^18^F-FDG could be detected as early as an hour after laser irradiation in human tumor xenografts in mice administered with NS by intratumoral injection[7]. Although these and previous results show promising applicability of ^18^F-FDG PET imaging to visualize and quantify the effect of nanoparticle-assisted photothermal therapy, it remains to be investigated whether changes in tumor uptake of ^18^F-FDG can be used as a prognostic marker of treatment outcome. Hence, this was the objective of our study.

## Materials and methods

### Nanoparticles

The NS were commercially obtained from NanoComposix, USA. The supplier reported using transmission electron microscopy (TEM) that the silica core of the NS was 119.7 nm ± 8.9 nm and that the total diameter of the complex was 151.3 nm ± 7.7 nm. The NS were functionalized with 5 kDa poly(ethylene glycol) with a zeta-potential of -27.3 mV; also reported by the supplier. The absorbance spectrum of the NS was measured in a aqueous solution using a Cary5000 UV-Vis-NIR spectrophotometer (Agilent Technologies) and the TEM image acquired using CM100 TEM (Phillips).

### Animal model

All animal experiments were conducted in accordance with an approval from the Animal Research Committee of the Danish Ministry of Justice (2012-15-2934-00064). Human neuroendocrine lung carcinoid cell line H727 (ECCAC, Salisbury, UK) were cultured in RPMI 1640+ GlutaMAX medium supplemented with 10% fetal calf serum and 1% penicillin-streptomycin (all Thermo Fisher Scientific) at 37°C and in 5% CO_2_. Cells were harvested by trypsinization at 80% - 90% confluence and resuspended in a 1:1 mixture of growth media and Matrigel (BD-Biosciences). Subcutaneous tumor xenografts were established in the left flank of 6 weeks old female NMRI nude mice (Taconic) by inoculation of ~ 10^6^ H727 cells dissolved in 100 μl mixture. The health of the animals was monitored every day and the tumor dimensions were measured with a caliper three times weekly. The volume was calculated as: volume = ½(length x width^2^). Animals had access to water and chow ad libitum at all times during the experiments (except for before PET scans where they were fasted overnight). When reaching the humane endpoints, the animals were euthanized by cervical dislocation.

### Biodistribution (ICP-MS)

Four animals with an average tumor volume of 103.5 ± 8.1 mm^3^ were injected intravenously with NS (280 μL of 5.0 x 10^10^ NS mL^-1^) (NanoComposix, USA) via the tail vein. 24 hours after injection the mice were sacrificed and blood, liver, spleen, kidney, lung, muscle and tumor were resected and stored at -80°C until being processed. To measure gold concentration in whole blood, blood (25 μl) was digested for 1 h at 65°C in HNO_3_ (150 μl), HCl (20 μl) and H_2_O_2_ (100 μl). MQ water (5 ml) was added to each tube. Tubes were weighed before and after addition of blood and digestive acids and water to accurately calculate dilutions. The digested blood solution was further diluted in HCl (2%) and subjected to ICP-MS measurements (ICAPq, Thermo Scientific, Hvidovre, Denmark) using ^197^Au as standard and ^193^Ir as internal standard (Sigma Aldrich, Brøndby, Denmark). Biodistribution of gold was assayed similarly to the blood. Here, tissue (50–100 mg) was dissected and weighed before being digested over night at 65°C in HNO_3_ (500 μl), HCl (50 μl) and H_2_O_2_ (300 μl). MQ water (10 ml) was added and after weighing, the samples were further diluted in HCl (2%) before ICP-MS measurements. The main instrumental operating conditions were as follows: RF power 1550 W and nebulizer gas flow 1,03 L min^-1^.

### Photothermal therapy

4–5 weeks after inoculation, animals were matched into three treatment groups based on tumor size: one receiving nanoshells and laser irradiation (NS group, 182 ± 51 mm^3^, *n* = 9), a control group receiving saline and laser irradiation (saline group, 163 ± 49 mm^3^, *n* = 9), and a control group receiving nanoparticles but no laser irradiation (sham group, 190 ± 53 mm^3^, *n* = 5). During all treatment procedures, the animals were anesthetized by breathing sevoflurane and their temperature was kept stable with a heating lamp or heating pad. The animals were injected intravenously with either 5.8 x 10^10^ NS mL^-1^ (240 μl) or saline (240 μl) via the tail vein. Approximately 24 hours after injection the animals were placed on a laser treatment platform and the tumors were irradiated with an 807 nm diode laser for 5 minutes using a laser intensity of 1.8 W cm^-2^ (beam diameter ~ 1 cm). Two animals injected with NS were euthanized immediately after laser irradiation because of burn blisters on the tumor and distress. However, they were still included in the thermographic assessment. After the treatment, the change in tumor volume (= ½(length x width^2^)) was followed by caliper measurements three times weekly with the humane endpoint defined as a tumor volume ≥ 1,000 mm^3^.

### PET/CT imaging

Animals were ^18^F-FDG PET/CT scanned immediately before they were injected with either NS or saline the day before treatment (baseline), and at day 1 after therapy. ^18^F-FDG was obtained from the daily productions for clinical use at Department of Nuclear Medicine & PET, Rigshospitalet, Denmark. Before each ^18^F-FDG PET/CT scan, the animals were fasted overnight and were anesthetized by breathing sevoflurane in the time from ^18^F-FDG injection and until after the PET/CT scan was completed. The animals were injected with ^18^F-FDG one hour prior to the PET scan (9.44 ± 0.99 MBq on baseline and 9.06 ± 0.91 MBq on Day 1; mean ± SD). The body temperature was kept stable using a heating lamp or heating pad. Static PET images were acquired 60–70 min post injection with an energy window of 350–650 KeV and a time resolution of 6 ns and CT scans were acquired using 360 projections, 65 kV, 500 μA and 400 ms (Preclinical PET/CT Inveon, Siemens). Acquired PET datasets were reconstructed using a 3-dimensional ordered subset expectation maximization (OSEM-3D) with maximum a posteriori (MAP) algorithm and CT-based attenuation correction (no. of subsets = 16, no. of OSEM-3D iterations = 2, no. of MAP iterations = 18, beta value = 0.053). Images had a voxel size of 0.4 x 0.4 x 0.8 mm^3^ and a resolution of approximately 1.2 mm at the center field of view. PET and CT images were co-registered and analyzed with Inveon Research Workstation software (Siemens PreClinical Solutions). Regions of interests (ROIs) were manually drawn on whole tumor regions from which the ^18^F-FDG uptake was quantified as mean percentage of injected dose per grams of tissue (%ID/g).

### Thermographic temperature measurements

During laser irradiation, the temperature at the surface of the tumor area was recorded using real-time thermographic imaging (FLIR T-440 camera) every 30 s. The images were analyzed using FLIR tools software to extract the maximum temperature on the tumor surface. All animals subjected to thermographic imaging in the study (both from treatment evaluation study and immunohistochemistry/autoradiography study) are included.

### Autoradiography and immunohistochemistry

Animals were treated with NS (*n* = 2) or saline (*n* = 2) and irradiation according to the above described treatment protocol. One day after, they were injected with ^18^F-FDG (~ 10 MBq) via the tail vein while being anesthetized by breathing sevoflurane. One hour after injection, the animals were euthanized and the tumors resected. The NS treated tumors were, however, too porous to separate completely from the skin. Tumors were immediately frozen by immersion in isopentane and embedded in tissue-tek. When it had solidified, the tumors were sectioned into ~9 μm slices. The sections were exposed on phosphorous films and imaged on a Cyclone plus imaging system (model C431200, Perkin Elmer) for visualization of the ^18^F-FDG intratumoral distribution. Thereafter the sections were dried overnight at room temperature and transferred to -80°C for further immunohistochemical analysis.

For immunohistochemistry, frozen tissue sections were equilibrated to room temperature for 30 minutes. Complete removal of tissue-tek was achieved by immersing the slides first in acetone at 4°C and then in HistoClear solution. Sections were then rehydrated through a series of ethanol solutions. Heat-induced epitope retrieval (HIER) was performed by submerging the slides in citrate buffer (20mM) at pH 6 and steaming at 100°C for 15 minutes in a microwave. Once equilibrated to room temperature and rinsed in running water, sections were immersed in K-PBS+tween20 for 5 minutes. Slides were kept in PBS until staining. The immunohistochemistry procedure was performed in a humidified chamber. Tissue sections were blocked first with peroxidase blocking solution for 10 minutes and then with BSA (2%) for 20 minutes. Primary antibody, anti-CD68 (1:200, #ab125212; Abcam), was diluted in BSA (2%) and incubated for 1 hour. The slides were then incubated with HRP-labelled polymer conjugated to secondary antibody (EnVision + System-HRP Labelled Polymer) anti-rabbit for 40 minutes. Slides were subsequently incubated with DAB (Liquid DAB+ Substrate System™, Dako; K3468) for 5 minutes at room temperature, then rinsed in PBS and later in running water for 5 minutes. Finally, the sections were counterstained with hematoxylin, dehydrated and mounted using the Coverslipper. Additionally, H&E staining was performed in some slides to allow observation of necrosis in the tissue. For this purpose, sections free of tissue-tek and rehydrated were stained with hematoxylin for 3 minutes, rinsed in tap water for 5 minutes and finally stained with eosin for 1 minute.

### Statistical analysis

The temperature elevation measured on the surface of the tumor with FLIR camera was compared with one-way ANOVA with Tukeys post-hoc test. Survival was based on tumor volumes ≥ 1,000 mm^3^ (humane endpoint), and curves were analyzed using Log-rank Mantel-Cox test. The mean ^18^F-FDG uptake ratios were compared with one-way ANOVA with Tukeys post–hoc test. Statistical analyses were performed using GraphPad Prism 6.

## Results

### Biodistribution of NS

**Fig 1:**
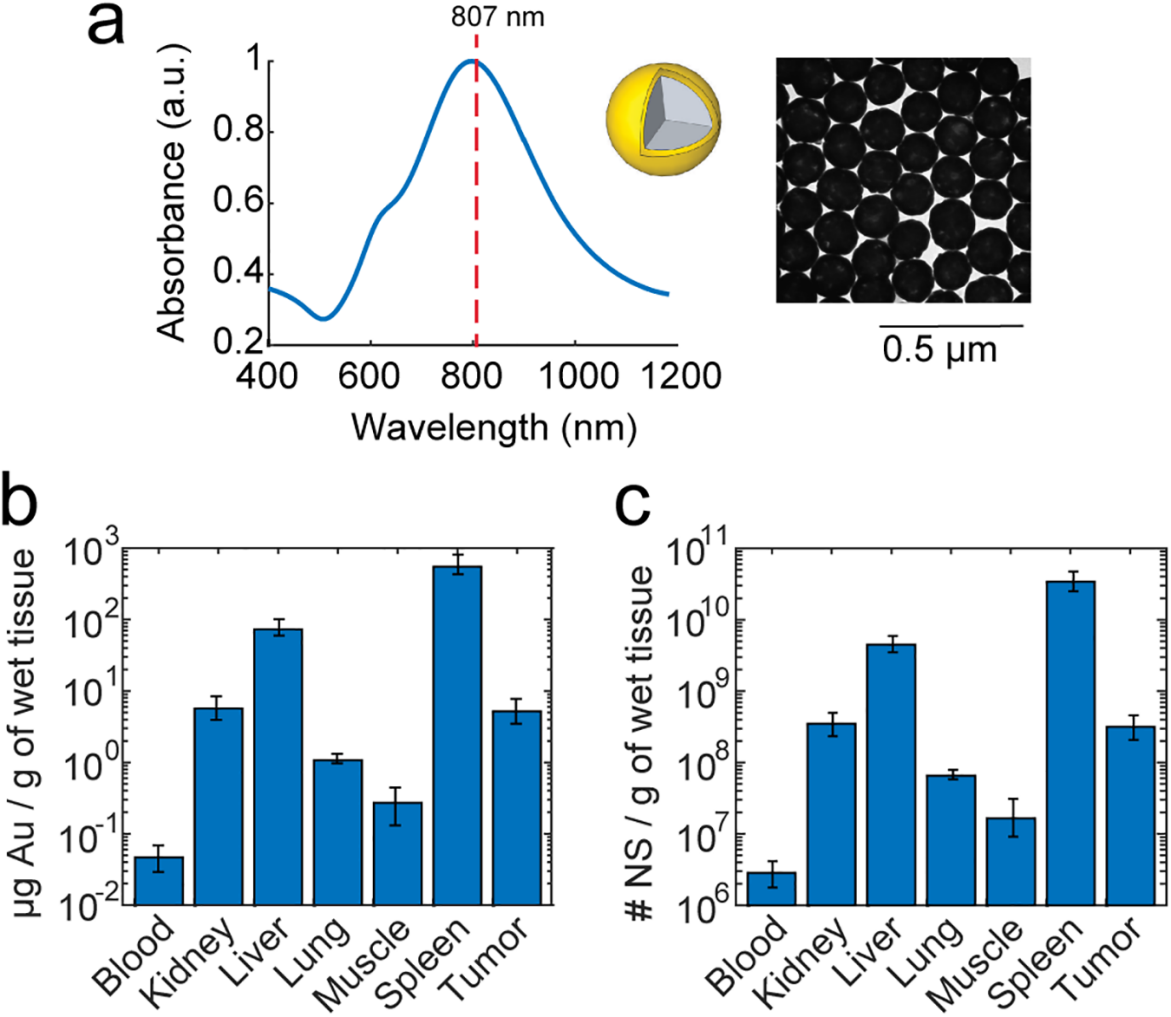
Photo-absorption properties and biodistribution of 150 nm NS. (a) Absorbance spectrum of NS measured in water using UV-vis spectrophotometry (*insert*: TEM image and illustration of a NS). The dotted line represents the wavelength of the NIR excitation laser (807 nm). (b) The mean Au content measured using ICP-MS in different tissues and (c) the corresponding number of NS measured 24 hours after intravenous injection. The biodistribution data is plotted on a log-scale to better resolve the Au content in tissues with very low uptake. Data shown is the mean ± S.E.M., *n* = 4.

As NS have been approved for clinical trials, image-based evaluation of their performance is highly relevant and hence they were chosen as the photothermal agent for this study. They exhibit maximum photo-absorption properties around 800 nm (see absorbance spectrum in Fig 1A) which is achieved by having a nanoparticle construct with a silica core of 120 nm in diameter surrounded by a 15 nm thick gold shell (see illustration and TEM image of NS in Fig 1A). We also recently published a study where the photothermal capabilities of these NS were characterized using both single particle and bulk assays[7]. The nanoparticles were functionalized with 5kDa poly(ethylene glycol) (PEG) to passivate the nanoparticle surface and prolong circulation time in the bloodstream. To confirm that the NS accumulate in H727 tumor xenografts in mice upon intravenous injection we first evaluated their biodistribution at the time point corresponding to laser treatment initiation. 24 hours after NS were injected intravenously via the tail vein, four animals were euthanized and the Au content in the blood, tumor, and different other tissues was measured using inductively-coupled-plasma mass spectroscopy (ICP-MS), see Fig 1B.

The uptake in the liver and spleen was substantially higher than in any other tissue which is in line with what is commonly reported in literature for nanoparticles in the size range of >100 nm[20,29]. Also, the blood showed a very low Au content suggesting that most NS were cleared 24 hours after injection. The number of NS in each tissue can be estimated from the measured Au content by considering that a NS of the above mentioned dimensions contains 1.66 x 10^−8^ μg Au. Based on the result shown in Fig 1C, the percentage of injected dose per gram tissue (%ID/g) was found to be 2.24 ± 0.8%ID/g (mean ± S.D.) in the tumor, which is consistent with values reported in literature for NS of similar size[1]. Lung and muscle, which should not take up nanoparticles from the blood stream, show accordingly relatively low Au content.

### Photothermal treatment of tumors using NS

For photothermal therapy, H727 tumor xenografts were established in the left flank of female NMRI nude mice. The tumors were allowed to grow for 4–5 weeks, at which point they had reached a volume of ~ 100–300 mm^3^, and were matched into 3 treatment groups: one receiving NS and laser irradiation (NS group; *n* = 9), a control group receiving saline and laser irradiation (saline group, *n* = 9), and a control group receiving NS but no laser irradiation (sham group, *n* = 5). The animals were injected intravenously with 240 μl of either 5.8 x 10^10^ NS mL^-1^ or saline via the tail vein. Approximately 24 hours after injection the animals were placed on a laser treatment platform and the tumors were irradiated with an 807 nm diode laser for 5 minutes using a laser intensity of 1.8 W cm^-2^. During laser irradiation, the temperature at the surface of the tumor area was measured using thermographic imaging.

**Fig 2:**
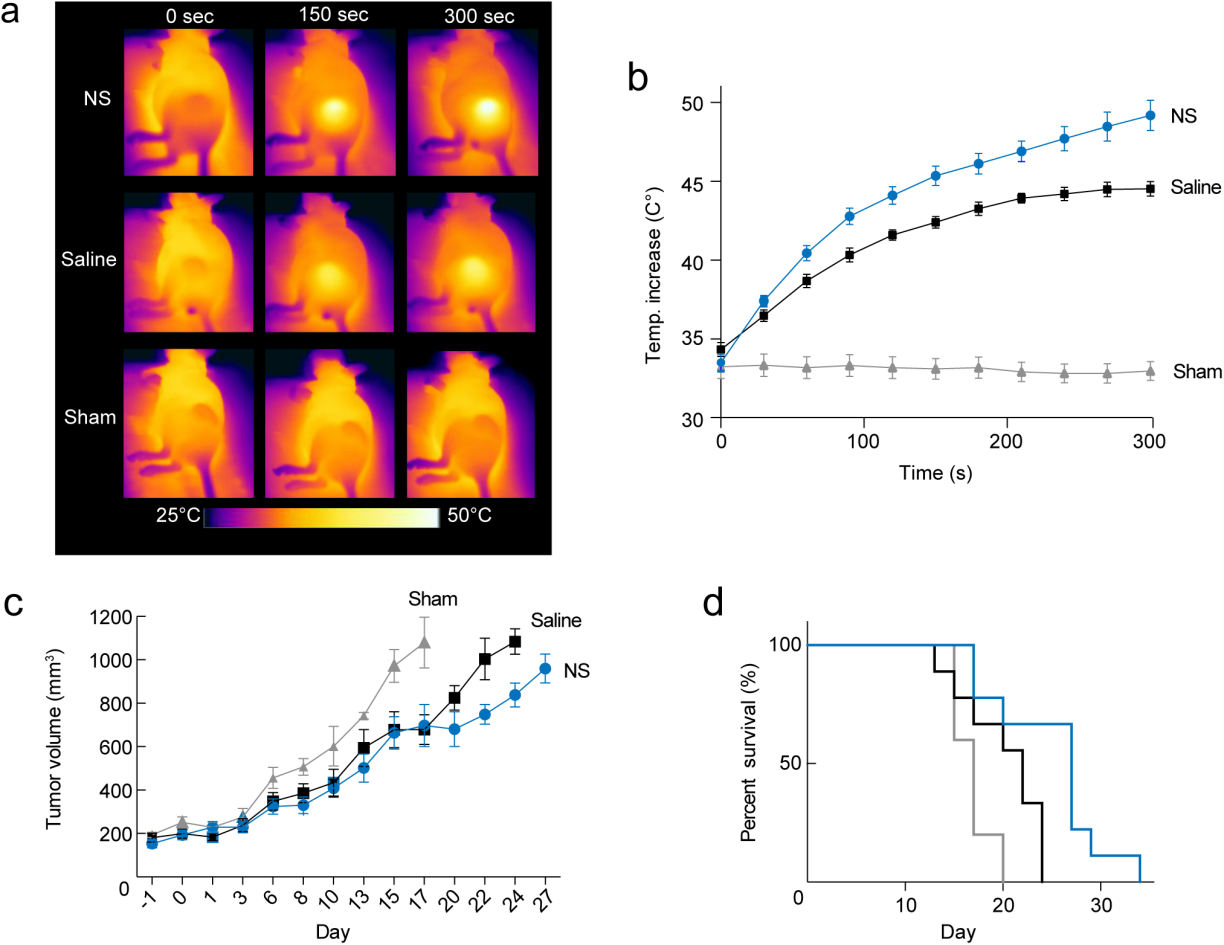
Evaluation of photothermal treatment. (a) Representative thermographic images showing the temperature at the surface of the tumor during 5 min treatment of animals from each group. (b) Mean temperature at the tumor surface as a function of time for all animals included in the study, i.e., PET and survival evaluation, immunohistochemistry, and autoradiography. (c-d) The tumors that were treated with NS and laser showed a delayed growth and improved survival compared to the saline and sham groups. In (b-d) data shown in blue circles/lines; *n* = 9 represents the NS group, data shown in black squares/lines; *n* = 9 represents the saline group, and data shown in grey triangles/lines; *n* = 5 represents the sham group. Data shown is mean ± S.E.M. Each growth curve in (c) is shown until *n* = 3.

Fig 2A and 2B show the temperature development in the tumors during the course of laser irradiation for the three different groups. After 5 min laser irradiation, the temperature at the tumor surface on average reached 49.2 ± 1.0°C (mean ± S.E.M.) in the NS group. In comparison, the average tumor temperatures reached 44.5 ± 0.5°C in the saline group and 33 ± 0.6°C in the sham group. Although the NS group experienced a significant larger temperature elevation (*p* ≤ 0.0001), it is clear that the laser light in itself was also unspecifically absorbed in the tissue as the temperature increased by *ΔT* ~10°C in the saline group over the course of irradiation.

After therapy, the change in tumor volume (= ½(length x width^2^)) was followed by caliper measurements until the tumor volume exceeded 1,000 mm^3^ where the animals were euthanized (see Fig 2C; each growth curve is shown until *n* = 3). In the NS group, the tumor growth was overall inhibited compared to the other two groups, although the treatment response within the group was found to be heterogeneous. In Fig 2D the survival curves of the groups were compared and were found significantly different (*p* < 0.01) with median survivals of 17 days for Sham, 22 days for Saline and 27 days for NS. The inhibited growth and improved survival of the saline group compared to the sham group suggest that the unspecific tissue heating observed contributes to the treatment effect.

### ^18^F-FDG PET imaging for early treatment response evaluation

**Fig 3:**
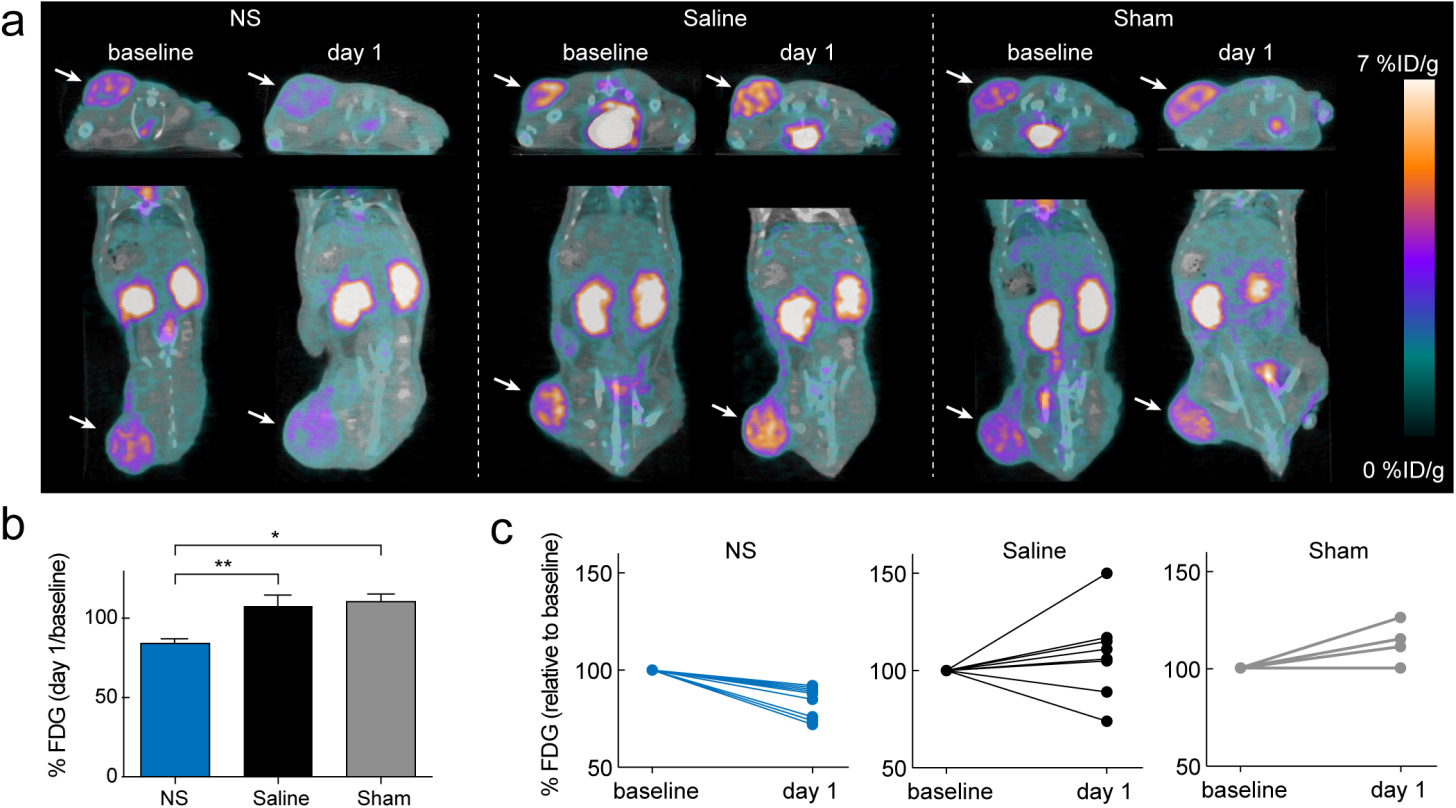
PET-based treatment evaluation. (a) Representative ^18^F-FDG-PET/CT images showing an animal from each group at baseline and day 1. Tumors are marked by white arrows. The ^18^F-FDG uptake is markedly reduced at day 1 compared to baseline for the NS treated animal. (b) The mean ^18^F-FDG uptake at day 1 relative to baseline for all three groups. * denotes *p* value < 0.05, ** denotes *p* value < 0.01 and data represents mean ± S.E.M. (c) The individual ^18^F-FDG uptake at day 1 relative to baseline for the NS group (blue; *n* = 9), saline group (black; *n* = 9), and sham group (grey; *n* = 5).

The animals were scanned with ^18^F-FDG PET/CT the day before (baseline) and at the day after treatment (day 1). Fig 3A shows representative transverse and coronal PET/CT images of the ^18^F-FDG uptake in all three groups at baseline and at day 1. By visual inspection it is clearly seen that the ^18^F-FDG uptake in the NS-treated tumors was markedly reduced at day 1 compared to baseline. For analysis, PET and CT images were co-registered and regions of interests (ROIs) were manually drawn on whole tumor regions. The ^18^F-FDG uptake in tumor was quantified as mean %ID/g and was found to be high and comparable between groups in the baseline scans (NS group: 3.9 ± 0.1%ID/g (mean ± S.E.M.), saline group: 3.6 ± 0.2%ID/g, and sham group: 3.6 ± 0.1%ID/g). The treatment response was evaluated by the reduction in ^18^F-FDG uptake after treatment. This was calculated as the ratio between the mean ^18^F-FDG uptake at day 1 and at baseline, denoted %FDG. Fig 3B shows that %FDG was significantly reduced in the NS group (= 84 ± 3%; mean ± S.E.M.) compared to the saline (= 108 ± 7%; *p <* 0.01) and sham (= 110 ± 5%; *p <* 0.05) groups.

**Fig 4:**
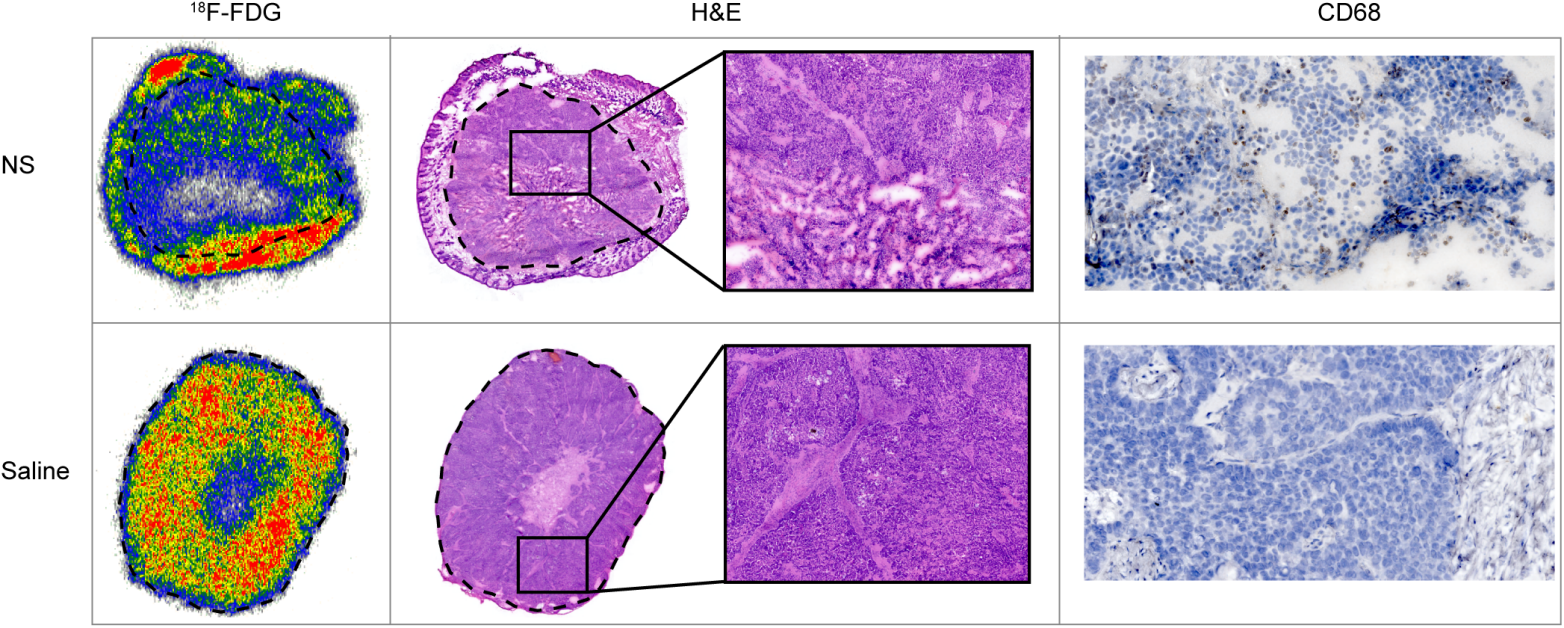
Autoradiography, H&E histological and immunohistochemical staining of a NS and saline treated tumor. Autoradiography of the ^18^F-FDG distribution in a NS and saline treated tumor. The dashed line marks the border between tumor and skin tissue. The NS treated tumor had a lower uptake of ^18^F-FDG than the saline treated tumor. From H&E staining of sections from the same tumors, the tissue in the NS treated tumor was found to be highly porous and necrotic whereas the saline treated tumor had a core with central necrosis but otherwise the tissue appeared normal. Finally, immunohistochemical staining with macrophage marker CD68 showed an increased level of macrophage infiltration in the NS treated tumor compared to the saline treated tumor.

We also plotted %FDG for each individual animal within each group, see Fig 3C. It is clearly seen that all animals in the NS group had reduced tumor metabolism at day 1 after treatment, although falling into two groups around ~90% and ~75% of their baseline value. In the saline group, two animals had a reduced tumor uptake of ^18^F-FDG at day 1 after treatment, probably caused by unspecific tissue heating. The rest of the animals in the saline group and all animals in the sham group had a similar or increased tumor uptake of ^18^F-FDG at day 1 after treatment. Moreover, to confirm the relationship between treatment-mediated tumor damage and reduced tracer uptake, we used autoradiography to compare the intratumoral distribution of ^18^F-FDG to hematoxylin and eosin (H&E) histological staining. Fig 4 shows autoradiography images of tissue sections of a NS and saline treated tumor, respectively. Compared to the saline treated tumor, a large region with very low ^18^F-FDG uptake was observed in the NS treated tumor. The corresponding H&E stainings are shown for the same tumors providing clear evidence that the regions with reduced ^18^F-FDG uptake had a high degree of tissue necrosis as well. Interestingly, we also observed a high ^18^F-FDG uptake in the skin of the NS treated tumor (outside the dashed line in Fig 4). This we speculate could be a consequence of biological processes related to wound healing and edema. In the tumor from the saline treated animal, reduced ^18^F-FDG uptake was only observed in a smaller central necrotic region, a common phenomenon in the H727 tumor model.

Also, to investigate the degree of treatment-induced inflammation that potentially can cause variation in ^18^F-FDG tumor uptake, we performed immunohistochemical staining for macrophage marker CD68 (see Fig 4). Overall, a higher level of macrophage infiltration was observed in the NS treated tumors compared to saline treated tumors, indicating that a NS-assisted photothermal treatment likely can induce an inflammatory response.

**Fig 5:**
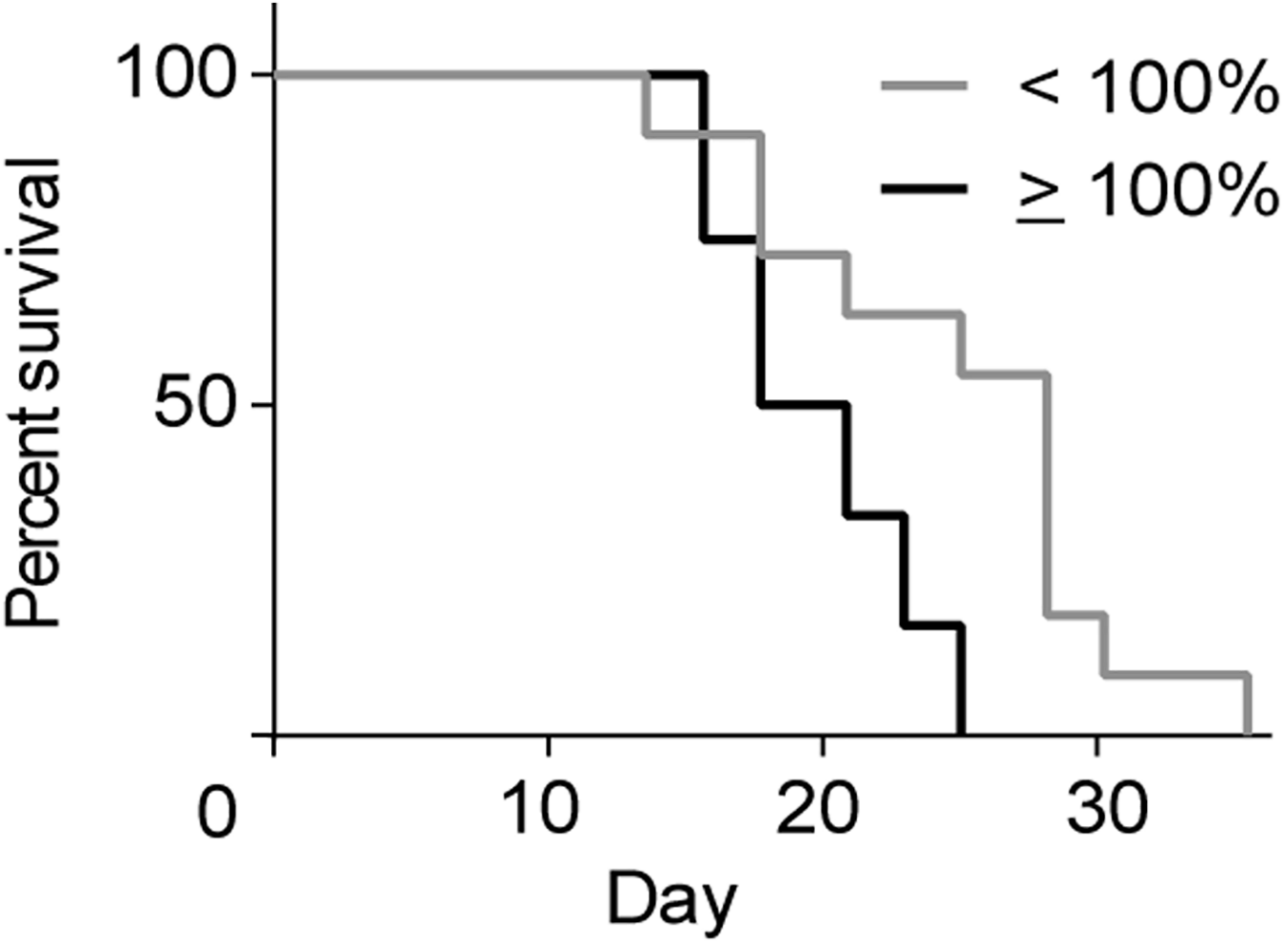
Stratification of animals based on their %FDG value. Animals with a %FDG lower than 100% (*n* = 11) had a significant (*p* value < 0.01) improved survival compared to animals with a %FDG equal to or above 100% (*n* = 12).

Finally, to see if the change in ^18^F-FDG uptake could be used to stratify the animals into groups representing responders and non-responders, all animals were divided into two groups representing: tumors with a reduction in %FDG and tumors with unchanged or increased %FDG after treatment. Fig 5 shows that stratification significantly differentiated survival between the two groups (*p* < 0.01) with median survival of 18.5 days for animals with %FDG ≥ 100% and 27 days for animals with %FDG < 100%.

## Discussion

Since the first use of nanoparticle-assisted photothermal therapy, it has been established with a wide range of heat-generating nanoparticles that the treatment can effectively damage or eliminate tumors in animals. It is also well-known that the therapeutic outcome of photothermal therapy is a combination of many elements.

First of all the treatment requires sufficient laser intensity in the tumor combined with nanoparticles capable of absorbing the light and efficiently convert this energy to reach hyperthermal temperatures. In literature temperatures of > 65°C are commonly reported for nanoparticle-assisted photothermal therapy but recently it was shown using MRI that a temperature of ~50°C is sufficient to inflict impaired tumor growth and that the effect can be enhanced by longer exposure times[11]. In our study it was evident that the NIR laser (operated with a typical dose compared to literature) in itself induced temperatures of ~45°C corresponding to a temperature elevation of *ΔT* ~10°C. Although this increase is on the same level as what is commonly reported in laser-treated control animals in literature [1,10,30,31], this temperature regime is well above physiological temperatures and can cause temporary or even irreversible cellular damage. Even though the NIR window represents a spectral region with high biological transparency, water molecules and chromophores such as hemoglobin and skin pigment melanin, that are abundantly present in the path of the externally applied laser, still have non-trivial absorption. Hence, to avoid burns and unintended tissue damage that cause pain and discomfort to the patient, the laser dose should be optimized, e.g. its intensity and duration, such that the therapy provides effective nanoparticle-mediated focal ablation while minimizing absorption in surrounding healthy tissue[31].

A second element known to affect the therapeutic outcome is nanoparticle accumulation in the tumor upon intravenous administration, which is the most relevant delivery route of nanomedicine in cancer therapy. Tumor uptake is also influenced by several factors, amongst these are the nanoparticle dimension and surface functionalization that determine how well the nanoparticles extravasate and circulate, respectively[29]. Many researchers with expertise in design and synthesis are working towards developing nanoparticles that experience high light-to-heat conversion at sub-100 nm dimensions, and with coatings that prevent early clearance from the blood[1,2]. Furthermore, researchers have developed radiolabeled or MR active theranostic nanoparticles that can be tracked real-time allowing the laser treatment to be temporally optimized based on the pharmacokinetic biodistribution[12,23–26]. A much debated question in literature is whether active targeting can be used to optimize nanoparticle accumulation in tumors in vivo. Although many studies show that targeting increases the intracellular uptake of nanoparticles in tumor cells, unfortunately little effect is seen on the overall tumor accumulation[32–34]. Beyond the physical and pharmacokinetic properties of the nanoparticles, the EPR effect can also vary a lot between tumor type which even further complicates a general optimization of nanoparticle design[35].

In this study we observed a fairly large variance in generated temperatures and a relative heterogeneous response (assessed by PET imaging and survival). In addition, the tumor growth in the treated animals was only partially inhibited and no animals had complete tumor removal. Intravenously delivered nanoparticles are often found to accumulate mostly in the periphery of the tumor and little in the center[11,27,36]. This is probably caused by high interstitial pressure and poor perfusion in the tumor core that impede nanoparticle diffusion far into the tumor microenvironment[37]. Combined with variation in NS uptake in tumors, this can easily be the source of non-uniform heat generation that can leave some parts of the tumor untreated and increase the risk of recurrence. Furthermore, in literature the treatment outcome has been shown to be highly dependent on the size of the tumor when treatment was initiated. For instance, in a study using ~1000 mm^3^ tumors only inhibited growth could be induced[30] whereas studies using tumors of sizes < 100 mm^3^ report complete resorption[14,16]. Based on this, photothermal therapy clearly has a higher efficacy in small tumors, however, the larger tumors are clinically more relevant. One way to improve the efficiency of treatment could be to raise the overall intratumoral temperature well above the threshold of irreversible damage, by, e.g., increasing the irradiation dose. As discussed above, this would also imply increasing damage to surrounding healthy tissue and thereby challenges photothermal treatment as a specific therapy with minimal adverse effects.

In the context of the discussed issues, there is a need for methods for early evaluation of treatment response. In this study, we found that ^18^F-FDG PET imaging was able to stratify responders from non-responders even though the treatment response in fact was heterogeneous and to some degree modest. Hence, we believe that PET imaging can provide valuable information for treatment evaluation and optimization. Finally, since ^18^F-FDG is not taken up by non-viable cells, one might have expected a larger reduction in uptake in NS treated tumors, considering the rather large volumes with severe tissue damage. Apart from specific uptake of ^18^F-FDG, the tracer also accumulates in inflammatory tissue and therefore treatment induced inflammation could contribute to the signal as well. There could be other PET tracers that are more sensitive to the response of photothermal therapy, however, the availability and clinical relevance of ^18^F-FDG, makes it a very strong candidate for future translational applications.

## Conclusion

In this study we showed that ^18^F-FDG PET could be used as a prognostic marker for the therapeutic outcome of nanoparticle-assisted photothermal therapy in human tumor xenografts in mice. Using histological staining and autoradiography, we confirmed that tumor areas with reduced ^18^F-FDG uptake coincided with regions of high cellular damage and necrosis. Furthermore, based on the reduced ^18^F-FDG uptake in tumors one day after treatment, we stratified all animals into two groups representing responders and non-responders, where the responders had significant prolonged survival. Early noninvasive imaging of treatment response using PET is a valuable tool in preclinical and clinical evaluation and optimization of cancer therapies. Based on the results of this study, we suggest that PET can also be used for guiding treatment planning of photothermal therapy and for early identification of non-responders for which the strategy should be changed.
